# Highly Configurable 100 Channel Recording and Stimulating Integrated Circuit for Biomedical Experiments

**DOI:** 10.3390/s21248482

**Published:** 2021-12-20

**Authors:** Piotr Kmon

**Affiliations:** Department of Measurement and Electronics, AGH University of Science and Technology, Al. Mickiewicza 30, 30-059 Cracow, Poland; kmon@agh.edu.pl

**Keywords:** biomedical recording, current stimulation, biomedical experiments, uniformity

## Abstract

This paper presents the design results of a 100-channel integrated circuit dedicated to various biomedical experiments requiring both electrical stimulation and recording ability. The main design motivation was to develop an architecture that would comprise not only the recording and stimulation, but would also block allowing to meet different experimental requirements. Therefore, both the controllability and programmability were prime concerns, as well as the main chip parameters uniformity. The recording stage allows one to set their parameters independently from channel to channel, i.e., the frequency bandwidth can be controlled in the (0.3 Hz–1 kHz)–(20 Hz–3 kHz) (slow signal path) or (0.3 Hz–1 kHz)–4.7 kHz (fast signal path) range, while the voltage gain can be set individually either to 43.5 dB or 52 dB. Importantly, thanks to in-pixel circuitry, main system parameters may be controlled individually allowing to mitigate the circuitry components spread, i.e., lower corner frequency can be tuned in the 54 dB range with approximately 5% precision, and the upper corner frequency spread is only 4.2%, while the voltage gain spread is only 0.62%. The current stimulator may also be controlled in the broad range (69 dB) with its current setting precision being no worse than 2.6%. The recording channels’ input-referred noise is equal to 8.5 µV_RMS_ in the 10 Hz–4.7 kHz bandwidth. The single-pixel occupies 0.16 mm^2^ and consumes 12 µW (recording part) and 22 µW (stimulation blocks).

## 1. Introduction

**Figure 1:**
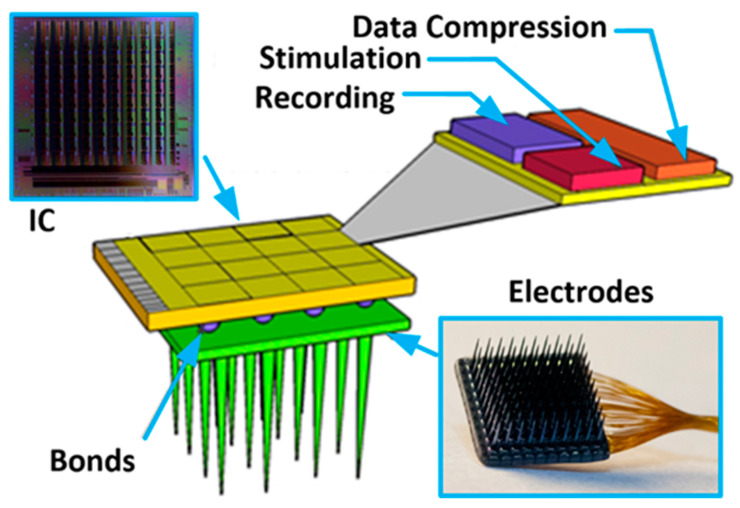
The conceptual idea of the implantable multichannel system for neurobiological experiments.

Existing technologies allow building systems comprised of sensors combined with electronics that can be used for different kinds of application (i.e., science, consumer market, military) [1,2]. Whenever such systems need to be very small, have large functionality, low power consumption, battery-less supply, and architecture allowing for multisite signal processing (see Figure 1), modern technologies are the only way to satisfy these requirements. These may be Micro-Electro-Mechanical Systems (MEMS) and Very Large-Scale Integration (VLSI) technologies for sensors and electronics production, respectively [3,4]. One of the contemporary technologies beneficiaries is also biomedicine which deals with experiments exploiting the electrical nature of neural-to-neural communication in living organisms. The main motivation of that experiments is the need for neural coding and processing cognition, finding cures for nervous system-related diseases, or developing prostheses for people with different disabilities [5,6,7,8,9,10,11,12]. These explorations are run as in vitro or in vivo experiments, and are realized with the use of electronic circuits that either record or/and stimulate neural activity. The common denominator for the mentioned experiments is the fact that the more active sites are in a single system (i.e., more inputs/outputs for recording/stimulation), the better the understanding of the observed phenomenon. Moreover, the systems’ spatial resolution is highly important as it allows for individual signal propagation monitoring (this parameter depends on the targeted experiments and can span from a single µm up to a few hundreds of µm). Next, a significant number of experiments require not only recording or stimulating neural activity, but also to have both of these functionalities and, what is more, to record different signal species (i.e., signals that vary in their amplitudes and frequency bandwidth). And finally, considering the in vivo experiments, one needs to also respect requirements regarding the wireless transmission of both energy and data. It becomes clear that a large number of active sites would be a source of a huge amount of data which, especially considering in vivo experiments, may pose a problem of the data bottleneck. Therefore, there is also a need to perform, directly on-chip data processing as much as possible to as to decrease the amount of valuable data, i.e., signal detection and classification, its conversion and data compression, etc. Having all of these in mind, one can see that neurobiologists expect highly functional systems comprising as many active sites as possible with large functionality, meaning that modern submicron or even nanometer technologies adaptation becomes one possible solution. Considering that the area dedicated to the single active site is imposed by a particular experiment, the area of the single conditioning system part needs to be reduced to allow for other functionalities to be implemented. Some solutions partially solve that problem, such as in [13,14,15,16], where the signal is pre-processed by the conditioning block, integrated with the electrode (with the use of the same fabrication process), and is further provided to the rest part of the system (additional conditioning analog circuitry and digital blocks). Moreover, Refs. [17,18] show a solution that mitigates mentioned problem with the use of 3D technology, i.e., the analog and digital parts are separated on individual dies, while their mutual connections are realized with the use of through silicon via (TSV). However, it should be pointed out that whenever the area occupied by the analog blocks becomes smaller, the problem that may negatively affect the whole neurobiological system, i.e., its performance or uniformity, rises [19,20]. Importantly, the parameters spread from channel to channel may introduce uncertainty in measured/generated signals (whether it comes to recording or stimulation) that may not be accepted, especially in experiments based on signal shape or its propagation parameters analysis. As a consequence, one needs to provide solutions to compromise on low area occupation of the analog part and its main parameters uniformity. Moreover, these solutions should not affect other systems’ parameters, such as power consumption or noise performance.

This paper aims to provide a possible solution that can be implemented whenever the abovementioned requirements need to be satisfied in the multichannel systems dedicated to different types of biomedical experiments. The proposed solution is a result of the author’s former works [21,22,23,24,25,26]. The paper is organized as follows. Section 2 provides information regarding the 100-channel integrated circuit (IC). Section 3 and Section 4 provide the design description of the recording and stimulating blocks, with an emphasis on their main parameter uniformity. Section 5 consists of the ICs’ parameters measurement results and neurobiological recording results, while Section 6 provides conclusions.

## 2. IC Architecture

**Figure 2:**
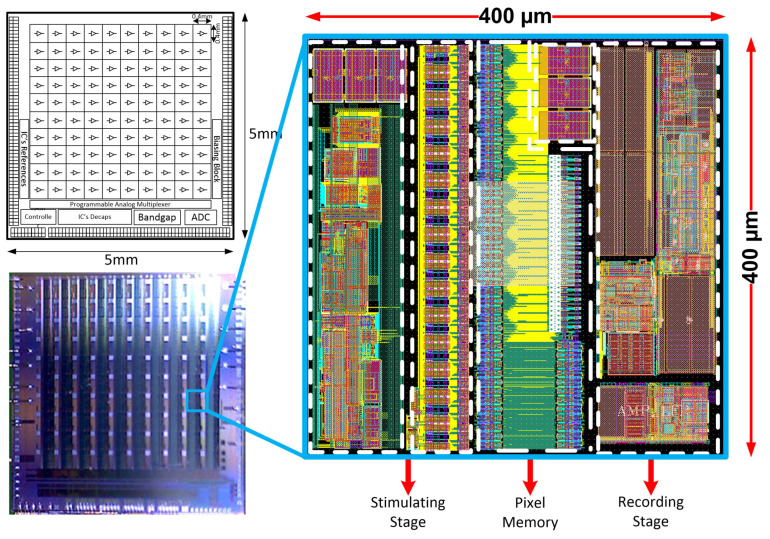
The NRS100 block idea, photograph of the PCB mounted IC, and the single pixels’ layout masks view.

The Neural Recording and Stimulating 100-channel Integrated Circuit (NRS100 IC) is composed of 10 × 10 pixels (each built of recording and stimulation blocks, and 36 × 9-bit memory), Successive Approximation Register (SAR)-based analog to digital converter (ADC), Random-Access Memory (RAM) controller, current/voltage user-controlled references, programmable analog multiplexer, and Low Voltage Differential Signaling (LVDS)- based receivers/transceivers (see Figure 2). The chip occupies an area of 5 × 5 mm^2^, whereas the single-pixel has a 400 µm pitch to meet 3D electrodes dimensioning (see Figure 1). Based on different biomedical experiments requirements, it was decided that the IC should allow one to record different biomedical signals, i.e., the lower corner frequency (LCF) should be set independently from channel to channel from approximately 1 kHz to below 1 Hz. The upper corner frequency (UCF) should also be independently controlled to allow for signals recording from single Hz up to a few kHz. Additionally, the particular IC’s recording channel should have the ability of its independent voltage gain control. Regarding the stimulation part of the IC, it was decided to provide the user ability to control in broad range stimulation parameters, i.e., stimulating pulses amplitudes, their duration, polarity, and even frequency. Therefore, each pixel has its memory which allows for defining pixel individual 30 stimulation patterns.

## 3. Recording Channel

The recording channel is dedicated to performing proper amplification and filtration of weak input biomedical signals to optimally utilize allowable supply voltage and to condition signals for following blocks, such as analog to digital converters. There are many excellent examples of integrated electronics dedicated to biomedical signals recordings [27,28,29,30,31,32,33,34]. These very often utilize additional techniques to overcome problems with noise and power consumption (such as chopping, sigma-delta conversion, ad digital feedback), and promoted results are outstanding. Here, the additional aspect is considered, i.e., main parameter uniformity from channel to channel, ICs’ functionality, and their area occupation that may be important whenever large functionality on a small area is considered [19,20].

**Figure 3:**
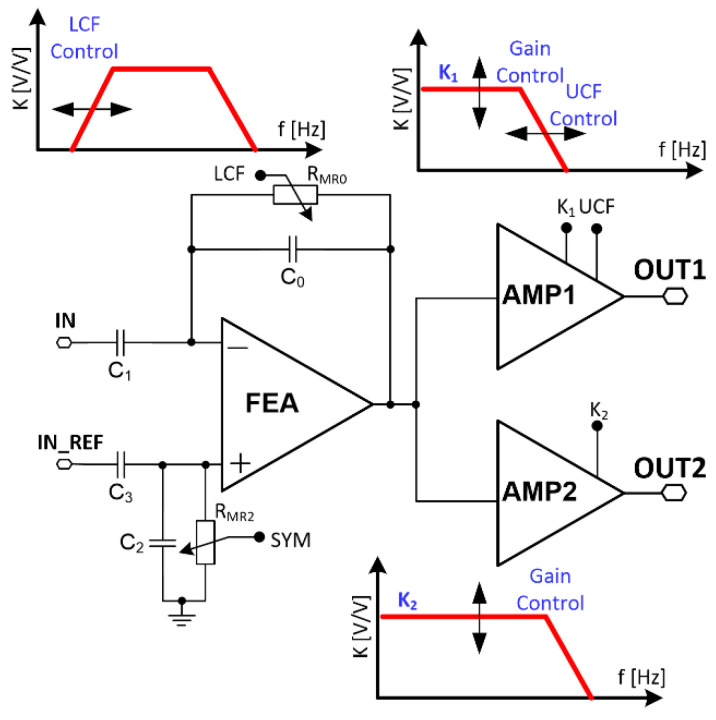
Recording channels’ schematic idea.

The biomedical signals differ in their amplitudes and frequencies bandwidth (see Table 1), and these parameters need to be taken into account in the complete recording channel design. It can be seen that whenever there is a need to record neurobiological signals with one circuitry, it is necessary to set different voltage gain and filters’ corner frequency. Therefore, a conventional, single path recording channel is not an efficient solution. If, for instance, LFP and AP signals are considered to be recorded simultaneously (see Table 1), it is necessary to set proper ADCs sampling frequency to avoid aliasing, and this results in unnecessarily high sampling frequency for LFP signals. Additionally, as the LFP signals are a few times higher in their amplitudes compared to the AP signals, one needs to set the correct voltage gain setting not to exceed the amplifier’s linear region. As a consequence, the voltage gain may be too small for AP signals, resulting in a higher ADC resolution requirement to properly reconstruct small AP signals. Having all of these in mind, the particular recording channel was divided into two individual conditioning paths, i.e., one for slow and the other for fast signal processing as shown in Figure 3. Both the corner frequencies and voltage gains can be changed in the particular conditioning signal path to address different input signal requirements.

Biomedical signals should be recorded differentially, as they are contaminated by common signals which are usually of much higher amplitudes than the input recorded signals are. Additionally, the front-end amplifier needs to comply with large input DC voltage offsets that are generated on the electrode interface reaching even hundreds of mV.

### 3.1. Voltage Gain

The recording channels’ mid-band voltage gain *K* is set by the front-end amplifier and following stages voltage gain, i.e., either the amplifier destined for slow or fast signals defined as *K*_1_ and *K*_2_, respectively:

(1) $$K=\frac{C_{1}}{C_{0}}\times K_{1/2}$$

In the given project the second amplifying stages are realized with the use of operational amplifiers working with the noninverting configuration. Here, the voltage gains are defined by the high poly-based *R*_1_, *R*_2_ resistors with the (*R*_1_ + *R*_2_)/*R*_1_ ratio. Finally, the voltage gain spread can be given in the following way:

(2) $${\left(\frac{\sigma_{K}\;}{K}\right)}^{2}={\left(\frac{\sigma_{C_{1}}\;}{C_{1}}\right)}^{2}+{\left(\frac{\sigma_{C_{0}}\;}{C_{0}}\right)}^{2}+{\left(\frac{\sigma_{R_{1}}R_{2}}{R_{1}\left(R_{1}+R_{2}\right)}\right)}^{2}+{\left(\frac{\sigma_{R_{2}}\;}{R_{1}+R_{2}}\right)}^{2}$$

In the given project the single-pixel was decided to be divided into three equal areas (recording, stimulation, and memory), resulting in an approximately 0.05 mm^2^ area dedicated to the recording stage. Additionally, bearing in mind that both the recording channel is composed of two amplifying stages and the feedback PMOS transistor forming the *R_MR_*_0_ resistance should be kept in the linear region, the voltage gain was set to 51 *v/v*. It was performed with the use of 52 unit MIM (metal-insulator-metal) capacitors each of capacitance and area 150 fF and 25.8 × 5.5 µm^2^, respectively. The *C*_1_ is composed of 51 unit capacitors, whereas the unit capacitance spread for a given process is *Ϭ_C_*/*C* = 0.034%. The area dedicated to the second amplifying stage was 110 × 110 µm^2^. This resulted in the AMP1 feedback composed of 24 unit resistors of *W* × *L* = 0.5 µm × 22 µm producing 50 kΩ unit resistance (*R*_1_ = 150 kΩ/300 kΩ, *R*_2_ = 900 kΩ), while the AMP2 was built of 44 unit resistances of *W* × *L* = 0.5 µm × 5 µm producing 11 kΩ unit resistance (*R*_1_ = 22 kΩ/44 kΩ, *R*_2_ = 440 kΩ). The single resistors’ spread value *Ϭ_R_*/*R* depends on the particular resistors’ value, and is equal to *Ϭ_R_*_1_/*R*_1_ = 0.11%, *Ϭ_R_*_2_/*R*_2_ = 0.49% (amplifier AMP2) and *Ϭ_R_*_1_/*R*_1_ = 0.11%, *Ϭ_R2_/R*_2_ = 0.19% (amplifier AMP1). Taking into account Equation (2), it results in recording channels voltage gain spread equal to 0.53% (fast signals) and 0.29% (slow signals). It can be seen that the voltage gain spread should be expected low, whereas its main spread contribution comes from the second amplifying stage composed of high poly resistors.

### 3.2. Lower Corner Frequency

The LCF of the recording channel is defined by the *R_MR_*_0_*C*_0_ constant that should be in the range from about 10 s down to 250 ms (considering the 0.1 Hz ÷ 1 kHz LCF tuning range). Assuming that the feedback capacitance *C*_0_ is equal to 150 fF, it is necessary to set the *R_MR_*_0_ in the range from 1.6 GΩ up to 10.6 TΩ. It can be seen that even for the highest required LCF, the *R_MR_*_0_ resistance still has to have an extremely high value requiring the use of nonpassive components. A natural alternative here is the MOS-based transistor working as a controllable resistor. Regarding MOS transistors’ effective channel resistance, this depends on a transistors’ operating region, i.e., either strong inversion (SI) or weak inversion (WI), and can be given as follows [35]:

(3) $$R_{SI}=\frac{1\;}{\beta (V_{GS}-V_{TH})}$$

(4) $$R_{WI}=\frac{1\;}{2n\beta \emptyset_{T}exp\left(\frac{V_{GS}-V_{TH}}{n\emptyset_{T}}\right)}$$

where *V_GS_* is the transistors’ gate-source dropout voltage, *V_TH_* is the transistors’ threshold voltage, *n* is a subthreshold slope factor, and *β = µC_OX_* (*µ*—represents carriers mobility, *C_OX_* is the oxide capacitance per transistor channel area), and *ϕ_T_* represents thermal voltage equal to 26 mV at room temperature.

There are two more aspects that must be taken into account while choosing the transistors’ proper operating region, i.e., its effective resistance tuning range and resistances’ spread from channel to channel. Thus, considering Equations (3) and (4), one can calculate the MOS channel resistance spread for SI and WI operating modes:

(5) $${\left(\frac{\sigma_{R_{SI}}\;}{R_{SI}}\right)}^{2}=\frac{\sigma_{VTH}^{2}\;}{{\left(V_{GS}-V_{TH}\right)}^{2}}+\sigma_{\beta }^{2}$$

(6) $${\left(\frac{\sigma_{R_{WI}}\;}{R_{WI}}\right)}^{2}=\frac{\sigma_{VTH}^{2}\;}{{\left(n\emptyset_{T}\right)}^{2}}+\sigma_{\beta }^{2}{\left(2n\emptyset_{T}exp\left(\frac{V_{GS}-V_{TH}}{n\emptyset_{T}}\right)\right)}^{2}\approx \frac{\sigma_{VTH}^{2}\;}{{\left(n\emptyset_{T}\right)}^{2}}$$

Considering the same transistors’ channel dimensions and that the *A_VT_* = 6.6303 mVµm and *A_β_* = 0.6114%µm for a given process, one would obtain *Ϭ_RSI_/R_SI_* = 0.14% and *Ϭ_RWI_/R_WI_* = 3.2% (one should keep in mind that *Ϭ_RSI_/R_SI_* value is produced for the best transistor operating conditions from the resistance spread point of view, i.e., the *V_GS_* is considered to be 1.8 V, whereas lowering the *V_GS_* would lead to *Ϭ_RSI_/R_SI_* getting worse). Moreover, it can be seen that one would get much lower MOS-based effective resistance whenever the transistor operates in the SI. However, assuming the same transistors’ channel dimensioning and that the PMOS transistor is used (higher channels’ effective resistance compared to NMOS transistor), one would need to obtain that to cover given frequency range the transistors’ *V_GS_* voltage needs to be changed only by 250 mV. On the other hand, utilizing the SI region, one would need to change the *V_GS_* voltage by almost 150 V, which is impossible. Therefore, the most promising way to satisfy the LCF tuning range is to use the WI transistors’ operating region and to compensate for its channel resistance spread from channel to channel through additional correction circuitry.

Finally, taking into account Equation (4), one can write the relation for the *LCF* of the recording channel:

(7) $$f_{LCF}=\frac{n\emptyset_{T}\;}{\pi }\frac{\beta exp\left(\frac{V_{GS}-V_{TH}}{n\emptyset_{T}}\right)\;}{C_{0}}$$

Next, considering Equation (7) a formula for the *LCF* channel to channel spread may be given:

(8) $${\left(\frac{\sigma_{f_{LCF}}\;}{f_{LCF}}\right)}^{2}=\frac{\sigma_{VTH}^{2}\;}{V_{TH}^{2}}+\sigma_{\beta }^{2}+\sigma_{C_{0}}^{2}$$

The *Ϭ_C_*_0_/*C*_0_ *LCF* spread contribution may be neglected as for *C*_0_ = 150 fF it is equal to 0.034% only. Here, taking into account the *LCF* control range, the PMOS transistor dimensioning was set to produce *Ϭ_β_/β =* 0.14%. Finally, one should expect the *LCF* spread from channel to channel to equal to 3.2% and this is dominated by the transistors’ threshold voltage spread.

### 3.3. Upper Corner Frequency

The UCF of the recording channel is different for AP and LFP stages (Table 1) and as formerly explained, it is here defined in the two recording channel blocks separately. Taking into account the AP stage it is set by the front-end amplifier, while the LFP is controlled by the amplifier AMP1 (Figure 1). The *UCF* is here defined by three factors: open and closed-loop gain (*K_VOL_* and *K_VCL_,* respectively), and the UCF *f_UCF_OL_* of the amplifiers’ working in the open loop gain:

(9) $$f_{UCF}=f_{UCF{\_}_{OL}}\left(1+\frac{K_{V\_OL}}{K_{V\_CL}}\right)$$

Considering Equation (9), one may write a relation for the UCF spread of the recording channel:

(10) $${\left(\frac{\sigma_{f_{UCF}}\;}{f_{UCF}}\right)}^{2}=\sigma_{fUCF\_OL}^{2}+\sigma_{KV\_OL}^{2}+\sigma_{KV\_CL}^{2}$$

**Figure 4:**
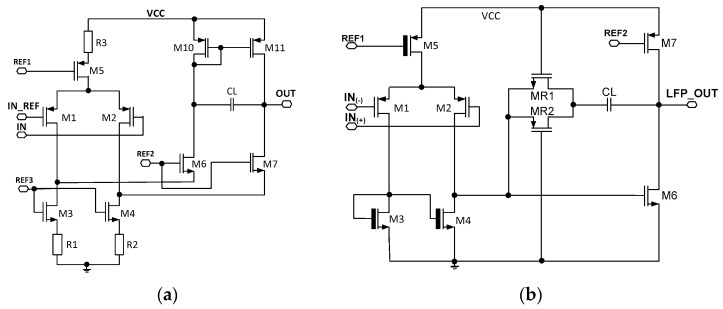
The schematic idea of the core amplifiers used in (**a**) front-end amplifier and AMP2, (**b**) AMP1 amplifier.

So as to estimate the *UCF* spread from channel to channel, a detailed analysis amplifiers architecture needs to be performed. In the following project, two core amplifiers are utilized and these work for front-end amplifier and amplifiers AMP1 and AMP2. The core amplifiers are presented in Figure 4. Since the three *UCF* spread factors analysis (Equation (10)) is very similar in both amplifying stages, only the front-end amplifiers’ UCF uniformity will be analyzed (Figure 4a).

Considering the first *UCF* spread component Equation (10), the open-loop *UCF* can be given in the following way:

(11) $$f_{UCF}=\frac{1\;}{4\pi r_{OUT}C_{L}}$$

where *C_L_* represents amplifiers’ loading capacitance (this is based on the MIM capacitor), and the *r_OUT_* is amplifiers’ output resistance (this can be considered as a resistance *r_ds11_* as resistance seen from amplifiers output into the *M*_7_ transistors drain is a few times higher). The resistance is proportional to the *V_A_* (first-order channel length modulation characteristic voltage) and inversely proportional to the *M*_11_ transistors’ current [35]:

(12) $$r_{dsM11}=\frac{V_{A}\;}{I_{M11}}$$

And

(13) $$V_{A}=V_{E}\frac{L_{M11}\;}{l_{a}}$$

where *V_E_* is a technological parameter and *l_a_* is a characteristic length given by [35]:

(14) $$l_{a}=\sqrt{t_{OX}\;d_{j}\;\frac{\varepsilon_{S}}{\varepsilon_{OX}}}$$

Finally, the open-loop gain *UCF* can be given:

(15) $$f_{UCF\_OL}=\frac{\sqrt{\;\frac{\varepsilon_{S}}{\varepsilon_{OX}}}\;}{4\pi V_{E}}\frac{I_{M11}\sqrt{\;t_{OX}\;d_{j}}\;}{L_{M11}C_{L}}$$

Having this relation, the open-loop amplifiers *UCF* spread may be evaluated:

(16) $${\left(\frac{\sigma_{f_{UCF\_OL}}\;}{f_{UCF\_OL}}\right)}^{2}=\sigma_{L_{M11}}^{2}+\sigma_{l_{M11}}^{2}+\frac{\sigma_{t_{OX}}^{2}\;}{4}+\frac{\sigma_{d_{j}}^{2}\;}{4}+\sigma_{C_{L}}^{2}$$

For a given process the ${Ϭ}_{t_{OX}}\;$ = 1.8% and the ${Ϭ}_{d_{j}}$ = 3% while the ${Ϭ}_{C_{L}}\;$= 0.003%.

Next, the open-loop gain of the front-end amplifier is given by:

(17) $$K_{V\_OL}=G_{m}r_{dsM11}$$

where *G_m_* is front-end amplifiers’ effective transconductance.

Here, the open-loop gain spread may be given:

(18) $${\left(\frac{\sigma_{K_{V\_OL}}\;}{K_{V\_OL}}\right)}^{2}=\sigma_{L_{M11}}^{2}+\sigma_{l_{M11}}^{2}+\frac{\sigma_{t_{OX}}^{2}\;}{4}+\frac{\sigma_{d_{j}}^{2}\;}{4}+\sigma_{G_{m}}^{2}$$

The transistors *M*_11_ channel length and its *I_M_*_11_ current were set to 1 µm and 300 nA, respectively. For a given technology, the *Ϭ_β_* = 2.81% that resulted in the total drain-source mismatches is equal to 1.8%. Having all the required data and neglecting *Ϭ_CL_,* the open-loop UCF spread is equal to 2.5%. Considering the amplifiers’ transconductance mismatches, it should be kept in mind that this is only the current *I_M1M2_* dependable parameter (input amplifiers’ transistors work in the weak inversion region resulting in *G_m_* = *I_M1M2_/nV_T_*). Therefore, the mismatches originate from the *M*_5_ transistors’ current mismatches. Here, for transistors’ *M*_5_ *W*/*L* = 10 µm/15 µm dimensioning and its current equal to 1.6 µA, the result is *A_β_* = 2.81%µm, finally producing 0.26% of *G_m_* spread. Having this in mind, the open-loop gain spread is equal to 6.8%. Furthermore, as it was previously shown, the closed-loop voltage gain spread is only 0.245. Ultimately, one should expect *Ϭ _fUCF_/f_UCF_* equal to approximately 7.2%.

## 4. Stimulating Channel

The electrical stimulation of either current, voltage, or charge impulses is often performed to trigger neuronal activity [36]. This may be required during the neuronal network explorations where, just after a predefined stimulation pattern, the recording is started to check neural network response [37]. Another stimulation application is its use during therapy where only stimulation pulses are generated with no need for recording [38]. Moreover, there are closed-loop implantable systems comprised of both stimulation and recording that are used as prostheses to restore some kinds of disabilities [39,40]. These types of implantable systems are the most complicated, as they need to operate quickly enough to provide proper operation of the controlled function. Therefore, these are comprised not only of recording and stimulation, but are also equipped with on-chip recorded signal classification, data compression, or wireless data transmission [41,42].

Here, it was decided to employ the current stimulation as compared to voltage and charge stimulation, since it is better in terms of impulses controllability, and area occupation. This, however, comes at the expense of higher power consumption.

**Figure 5:**
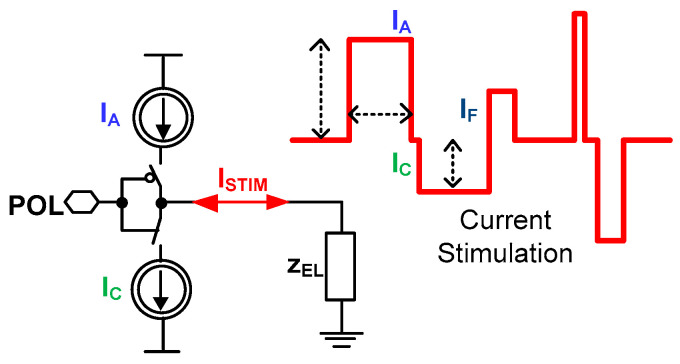
The conceptual idea of the current stimulator.

In the simplest stimulation form, there are only a few impulses required to perform effective stimulation (Figure 5). Often during the current stimulation, two types of current pulses are generated one after another, which is motivated by the experiment safety. The first pulse is an anodic current *I_A_* that triggers neuronal action, while the second current pulse is a cathodic current *I_C_* responsible for extracting charge provided by the anodic pulse. The charge extraction is necessary due to stimulation safety, i.e., any disproportions in these two currents lead to the stimulation electrodes’ built-in potential generation, which finally may be a reason for stimulated neuron death. Another important stimulation aspect is to perform stimulation in a way that allows fast switching from stimulation phase to recording phase not to miss any neural activity already triggered. There are different approaches to minimize that phase switching time [19,43,44,45,46]; however, the most promising one is generating a short current pulse *I_F_* just after the stimulation phase [47]. Additionally, there are experiments exploiting different current stimulation patterns to either code the information provided to the neural networks or to make the stimulation more efficient [48]. The most popular current stimulation applications with their main parameters are shown in Table 2.

Bearing all of these in mind, it can be seen that current stimulation should have the possibility of controlling not only I_A_ and I_C_, but also both the I_F_ current and timing relations of current stimulation particular phases.

**Figure 6:**
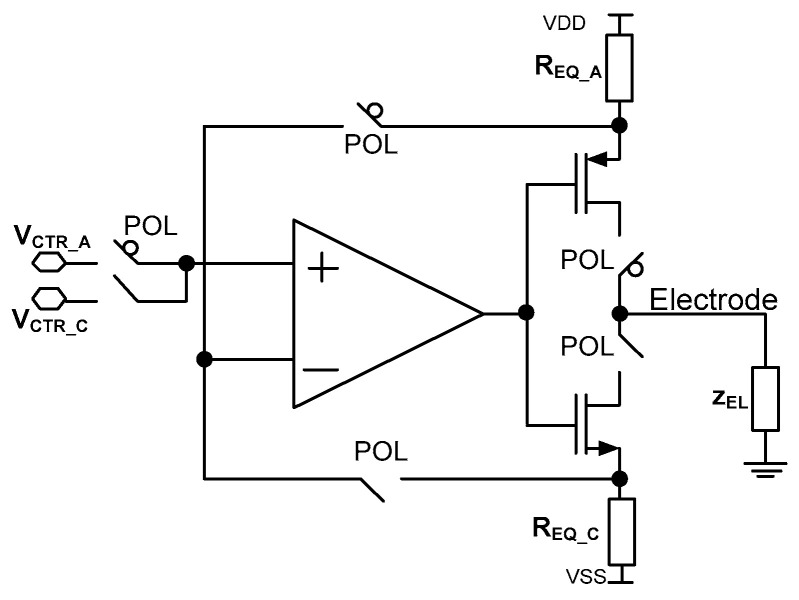
Current stimulator schematic idea.

There are many known solutions to the current stimulators’ architectures. The simplest one is based on two current sources composed of complementary PMOS and NMOS transistors. The other architectures exploit current sources’ cascading, capacitor-based memory for opposite stimulation current generation or adiabatic stimulation [48,54,55,56]. Here, a current stimulator architecture is proposed [57], allowing for both precise current control and current stimulation pattern generation for each of the stimulation current channels individually. This functionality is obtained thanks to the in-pixel RAM-supported programmable operational amplifier-based current source. Additionally, to achieve both, lower power consumption, area occupation, and to provide precise cathodic and anodic currents control, it was decided to use an operational amplifier current source (Figure 6).

**Figure 7:**
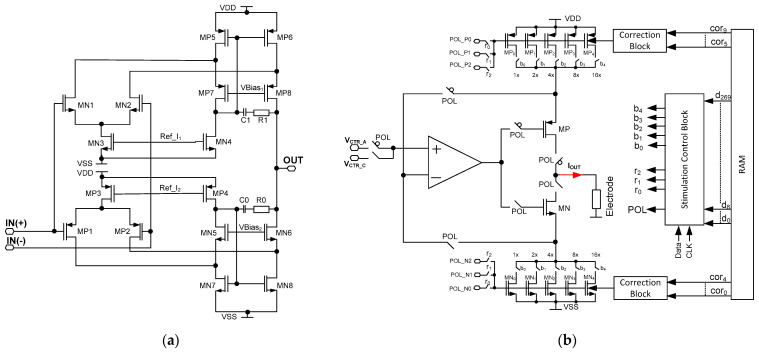
The schematic idea of the current stimulators’ core amplifier (**a**) and schematic idea of the programmable current stimulator (**b**).

The stimulator idea is based on the proper selection of two parameters, i.e., voltages *V_CTR_C_*/*V_CTR_A_* and resistances *R_EQ_C_*/*R_EQ_A_,* where *V_CTR_C_* and *V_CTR_A_* are voltages provided to the amplifiers’ input during one of the stimulation phases (cathodic or anodic current generation, respectively, controlled by the POL signal), while *R_EQ_C_*/*R_EQ_A_* is equivalent resistance during cathodic/anodic phase (Figure 6). To save power and silicon area, there is only one operational amplifier used that is built of rail-to-rail input stage (Figure 7a).

Considering the generated stimulation currents *I_ST_* spread, this depends mainly on two components, i.e., amplifiers voltage offset *Ϭ_VCTR_*/*V_CTR_* and resistance *R_EQ_* spread *Ϭ_REQ_*:

(19) $${\left(\frac{\sigma_{I_{ST}}\;}{I_{ST}}\right)}^{2}={\left(\frac{\sigma_{V_{CTR}}\;}{V_{CTR}}\right)}^{2}+{\left(\frac{\sigma_{R_{EQ}}\;}{R_{EQ}}\right)}^{2}$$

In the given project, the *R_EQ_* is composed of resistor bank *R* based on MOS transistors operating in weak inversion. Based on the different requirements of current stimulators (Table 2), it was assumed the current controllability for a given range should be performed with the step at approximately 5%. Therefore, the resistor banks are built of five binary-scaled MOS transistors which result in the current step control of 1/32 resolution. A single MOS based resistance is built of a PMOS or NMOS transistor with channel dimensions of *W*/*L* = 2 µm/1 µm or *W*/*L* = 1 µm/2 µm respectively. Then, having checked at Equation (19) the unit resistance spread should be approximately 3.2%. Therefore, the overall resistance spread is expected to be *√n* × 3.2%, where *n* represents unit resistances connected in parallel. Considering the *V_CTR_* voltage spread only, the voltage spread of the amplifier should be taken into account as the *V_CTR_* voltage is common for the whole IC. In a given operational amplifier architecture the MP1/MP2 and MN1/MN2 transistor should be taken into account as main voltage offset contributors. Their dimensioning is *W*/*L* = 50 µm/0.75 µm (MP1/MP2) or *W*/*L* = 4 × 50 µm/0.75 µm (MN1/MN2), and for *V_CTR_* = 200 mV, one should expect *Ϭ_VCTR_*/*V_CTR_* not higher than 0.4%.

The final stimulation block architecture is shown in Figure 7b. Here, the aim was to both cover different stimulation current ranges with their uniformity being no worse than 5%, and to allow for different stimulation patterns generation. Therefore, besides the resistor bank controllability of 1/32 step, the additional correction that is here used is based on controlling the MOS-based resistor bulks.

The current stimulator is also equipped with a stimulation control block that is a cyclic register, responsible for providing 1 out of 30 RAM stored channel individual settings. These define (Figure 7):

- current range (*r*_0_, *r*_1_, *r*_2_ outputs for selecting *POL_P/POL_N* voltages);
- current polarity (*POL* output for selecting either cathodic or anodic current);
- current value (*b*_0_ ÷ *b*_4_ outputs for defining the *R_EQ_* resistance value).

**Figure 8:**
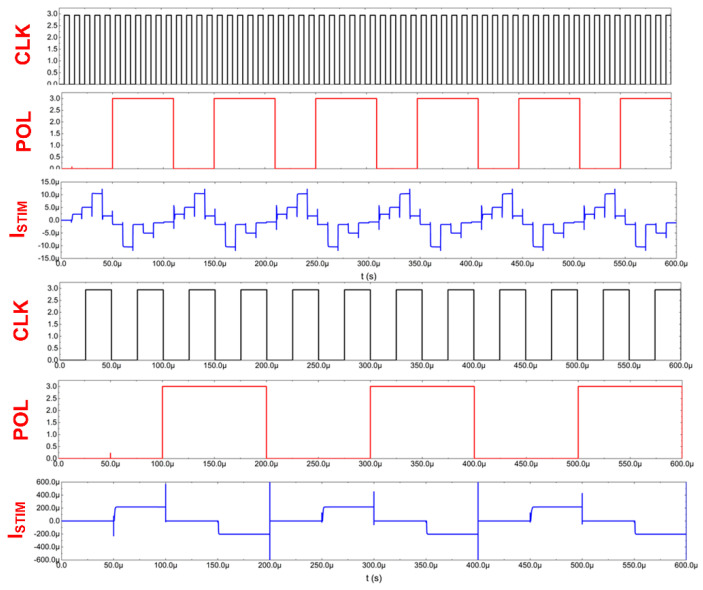
Current stimulators’ exemplary signals.

Thanks to the stimulator architecture, it is, therefore, possible to define 30 different stimulating currents that may be individually varied in terms of their timing parameters or current settings, as exemplarily shown in Figure 8.

## 5. Measurement Results

The NRS100 IC is designed in the CMOS 180nm process, occupying an area of 5 × 5 mm^2^, whereas the single-pixel occupies 400 × 400 µm^2^. The chip operation was verified by both its main parameters characterization and its application in neurobiological experiments. In total, the three ICs were verified.

The given paper focuses a lot on the IC’s main parameters uniformity that obliges to verify also a measurement systems’ precision. Here, the voltmeter and ammeter were used to measure recording and stimulating blocks, respectively. All measurements were carried out with the use of a LabVIEW environment.

The NRS100 recording channels verification was carried out with the help of the NI-USB 6351 multipurpose card. The voltage measurement accuracy was calculated based on the card datasheet, which is there defined by three uncertainty contributors: gain error, offset error, and noise uncertainty. Considering a voltage range used of ±0.5 V, these are 68 ppm, 17 ppm, and 26 µV_RMS_, resulting in approximately 0.5 mV absolute accuracy if a noise level of 36 and no averaging are considered. Taking into account that recorded signals are in the range of 100 mV ÷ 400 mV, this directly translates to a measurement error in the range of 0.5% ÷ 0.125%. Regarding the current stimulator verification, it was performed with the use of the Keysight HP34401 multimeter. Here, the current measurement error is defined by 0.005% of reading (4.5/30/400 µA stimulator current ranges) + 0.01% of range (10 mA), resulting in the measurement offset of 1 µA and measurement precision of 0.005%. In general, as shown further in the measurement results, these uncertainties are much lower than the results obtained, and may be omitted.

### 5.1. NRS100 Main Parameters Characterization

**Figure 9:**
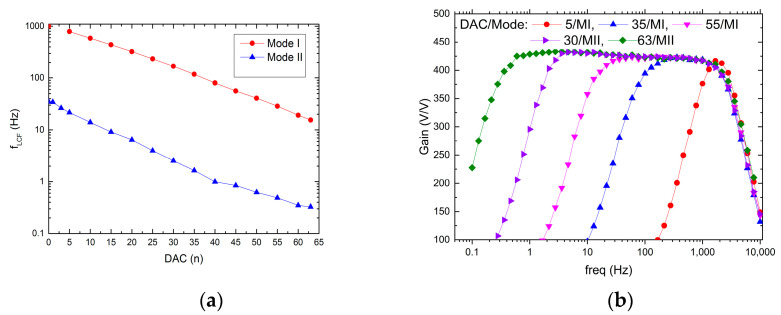
LCF control range (**a**) and exemplary frequency responses for different DAC settings (**b**).

**Figure 10:**
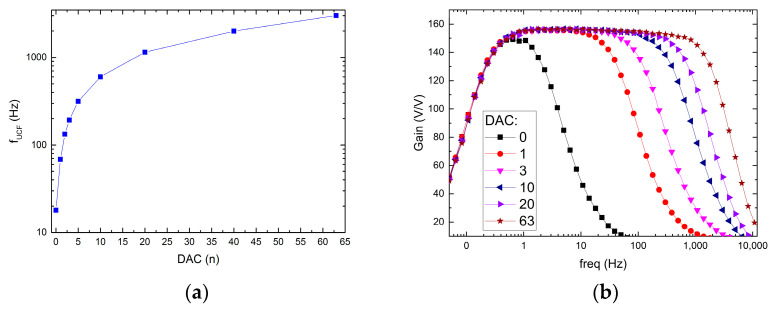
UCF control ranges (**a**) and exemplary frequency responses for different DAC settings (**b**).

The possible recording channel configurations are shown in Figure 9 and Figure 10. It can be seen that the LCF can be controlled in one of two ranges, i.e., in the 10 Hz ÷ 1 kHz range, or the 300 mHz ÷ 30 Hz range. Importantly, thanks to the fact that the particular pixel is equipped with its 6-bit DAC, the LCF may be tuned individually. The UCF can be controlled globally in the 20 Hz ÷ 3 kHz range. These results show that the thick oxide transistors usage in the second stage of the recording path (AMP1) (see Figure 4b) allowed for the decrease in current supplying the amplifier down to a few pA in each of the recording channels.

**Figure 11:**
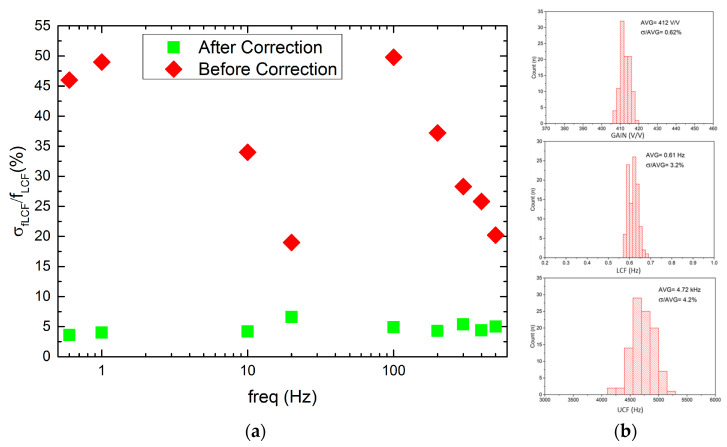
LCF spread from channel to channel before and after correction for a given LCF setting (**a**) and voltage gain, LCF, and UCF histograms (**b**).

Regarding the LCF uniformity (see Figure 11), it can be seen that it worsens whenever one starts to decrease in LCF, i.e., the Ϭ_fLCF_/f_LCF_ for 20 Hz and 600 mHz equals 17% or 45%, respectively. This is a result of LCF controlling DAC which is based on the weighted current mirror. Importantly, the LCF spread may be minimized down to 5% by the individual recording channel correction. The voltage gain and UCF spread are equal to 0.62% and 4.2%, respectively, which is in agreement with former calculations.

**Figure 12:**
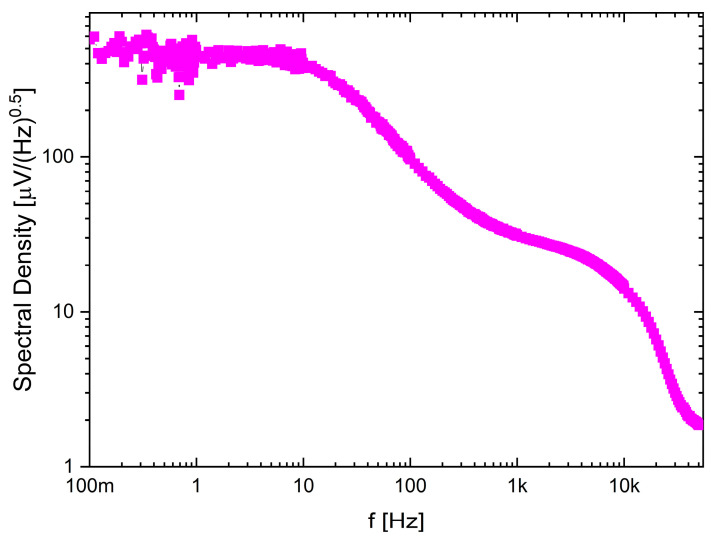
Noise spectral density for one of the selected recording channel settings.

The recording channel noise measurements (see Figure 12) show how the noise characteristic may be shaped by the frequency bandwidth selection. Here, the LCF was set to approximately 10 Hz, while the UCF to 4.7 kHz and it can be seen that the 1/f noise is mitigated below LCF.

**Figure 13:**
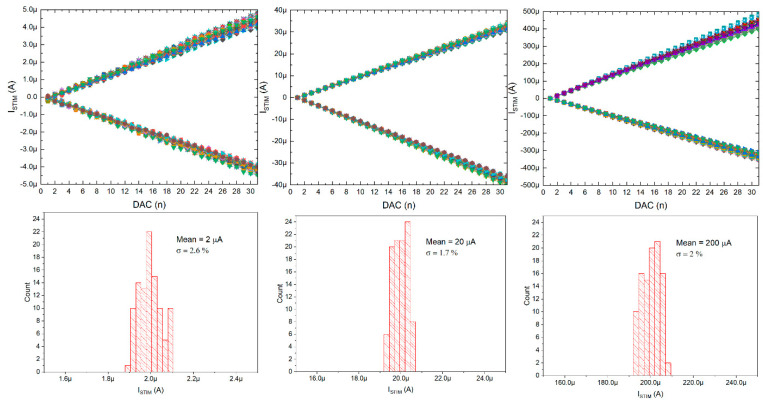
Current stimulators’ particular current ranges controlling range with inset pictures of histograms for particulars’ current correction (all 100 stimulation channels are here given).

The current stimulators were also verified (only static verification was conducted, these were not used during the neurobiological experiments), and it can be seen that the stimulating currents can be controlled with 32 equal steps in three different current ranges: 0 ÷ ±5 µA, 0 ÷ ±25 µA, and 0 ÷ ±450 µA (see Figure 13). These current ranges can be changed individually, as the POL_N/POL_P voltages are globally controlled by six 6-bit DACs located below the chip matrix. Noticeably, thanks to the in-pixel individual correction, the uniformity of the stimulating current can be as low as 2.6%.

The chip was also verified in terms of other parameters that are listed in the comparison Table 3. The author intended to compare his work to those designs that are fabricated in similar process nodes (i.e., 180 nm or 130 nm) to show that even such an old process may allow for developing ASIC with a large functionality and good performance. Only those designs with multichannel architecture and being comprised of recording and stimulation were taken into account. It can be seen that the NRS100, compared to other designs, is very attractive in terms of its main parameters broad and precise tuning ability, i.e., both corner frequencies, as well as stimulating currents. Importantly, even the single-pixel functionality is expanded, compared to other designs; it occupies a low area, and thanks to its architecture main parameters spread from channel to channel are significantly mitigated. The stimulating current programmability, recording channels power consumption of 12 µW, input-referred noise of 8.5 µV_RMS_, and individual main parameter correction make the proposed chip a good candidate for a variety of neurobiological experiments.

### 5.2. Neurobiological Experiments

**Figure 14:**
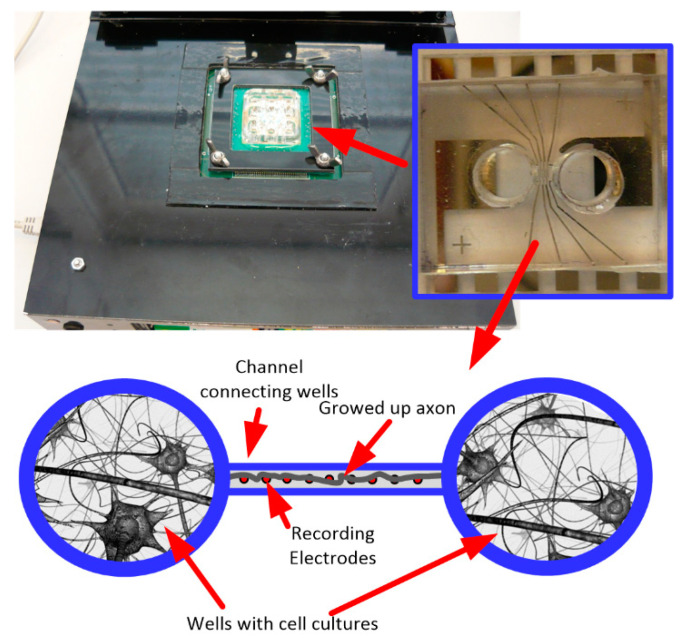
The photo of the 256 channel in-vitro recording platform with inset pictures of the specially developed multielectrode arrays used in the experiment, and figurative experiment description.

**Figure 15:**
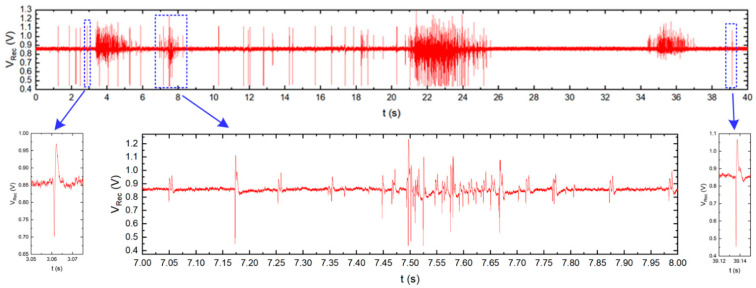
Typical neurobiological signals recorded by the chip presented. Top: signal recorded by one electrode located inside the microchannel. Bottom: enlarged single action potentials (**left** and **right**) and burst activity (**center**). In the burst, several signals from different axons grown through the microchannel can be distinguished due to different signal amplitudes and signal shapes.

The presented IC was also used in the neurobiological experiment performed in collaboration with Qwane Biosciences SA, Lausanne, Switzerland, that conducted extensive research under the NEUROACT project [58] The idea of this experiment was to produce a first feasibility study of the measure of neuronal signals propagation speed along isolated axons. The motivation of this experiment was to find a way that allows studying the propagation characteristics of neuronal signals along axons, as the impairment of axonal propagation is often a consequence of neurodegenerative diseases. This approach could lead to a platform for drug screening applications whereby neuronal protection or recovery could be monitored by the propagation speed along the axons. Therefore, to perform this experiment, the 256 channel in-vitro recording platform [59] was adapted to a specially developed Micro-Electrode Array (MEA) device (see Figure 14)—the few pixels were combined with existing recording electrodes, and the exemplary recordings are presented. The MEA is composed of two small culture wells measuring 3 mm in diameter, connected by a microchannel (10 µm wide, 3 µm high, and 1 mm long), allowing only neuronal axons to cross it. The recording electrodes (10 µm × 50 µm) are located at equidistant positions along the microchannel and not in the culture chambers as they typically are in MEA devices. The experiment may be started just after the neurons establish their well-to-well connection with the axon, which takes approximately 10–14 days [60,61]. The Figure 15 shows the neurobiological signals recorded during this experiment. Compared to standard MEA devices, obtained single spike amplitudes are much larger in our chip configuration (up to 700 µV instead of typical 50 µV ÷ 100 µV), which is due to the small volume of the microchannel in which the axons are located. As can be seen in the recording, different signal amplitudes are recorded during the experiment. These different signals correspond to different individual axons crossing the microchannel.

The comparison of the signals recorded at the different electrodes, i.e., at both ends of the microchannel, allows for the definition of the time of signal propagation through the microchannel, and the calculation of the propagation speed of the spikes along the axons.

## 6. Conclusions

In this paper, the design, measurement, and experiment results of the NRS100 chip are presented. The proposed architecture composed of both recording and stimulating functionalities supported by blocks responsible for main parameters correction, configuration, and programmability proves it may be highly efficient in a variety of biomedical experiments. Particular channels’ components spread influence is shown that allows one to define limits in area minimization of such systems.

It can be seen that, thanks to the different techniques employed in the presented design, it was feasible to develop a chip that has both a large functionality (ability to record different neurobiological signals, broad and precise multichannel programmable current stimulation) and good performance (i.e., low power consumption, small area occupation, low input-referred noise, low channel-to-channel parameters spread). These techniques are mainly:

- recording channels’ architecture (two followed stages with individual gain and bandwidth control);
- stimulating channels’ architecture (anodic/cathodic single based amplifier current source individually controlled);
- correction circuitry (in-pixel local DACs supported by global DACs located out-of-the pixel matrix, in-pixel RAM).

However, whenever more than hundreds of channels are required, the ASIC would need to be equipped with data compression, and therefore modern processes would need to be used. However, presented approaches could also be used.

## Figures and Tables

**Table 1:** Parameters of the typical biomedical signals [16].

Biomedical Signal	Amplitudes	Frequency Band
Local Field Potentials (LFP)	10 µV ÷ 5 mV	1 ÷ 500 Hz
Action Potentials (AP)	10 µV ÷ 500 µV	300 Hz ÷ 7 kHz
EEG	1 µV ÷ 10 µV	<1 ÷ 100 Hz
ECG	1 mV ÷ 10 mV	5 ÷ 500 Hz
EMG	100 µV ÷ 10 mV	20 Hz ÷ 1 kHz

**Table 2:** Typical current stimulation parameters [49,50,51,52,53].

Application	Stimulation Current [µA]	Duration [µs]
Cortex	400	500
Retina	100	27–1500
In vitro neural networks	1 ÷ 10	20 ÷ 1280
Deep Brain Stimulation	200 ÷ 2000	60 ÷ 120
Spinal Cord	500	60 ÷ 1000

**Table 3:** Performance summary and comparison table.

Specification	This Work	JSSC’14 [30]	JSSC’17 [32]	JSSC’21 [27]	TBCAS’16 [33]
Technology (nm)	180	180	130	180	180
Supply (V)	1.8/3.3	1.8	1.2/2.5/3.3	1/3	1.5/5
Neural Recording					
Area/ch. (mm^2^)	0.053	0.45	0.011	0.66	0.56
Power/ch. (µW)	12	57.7	0.63	2.5	5.5
Bandwidth (Hz)	(0.3 ÷ 1k)–(20 ÷ 3k) LFP (0.3 ÷ 1k)–4.7k AP	(0.1 ÷ 10) –(0.8 k ÷ 7k)	0.1–500	200–9k	0.25–250
Voltage Gain (dB)	43.5/52	41–61	Direct ADC w/o amplifier	27.6–50	Direct ADC w/o amplifier
Controllability	In-pixel LCF (6–bit), UCF (6–bit), Voltage Gain	Global LCF, UCF, and Voltage Gain	N.A.	Voltage Gain	UCF
Noise (µV_RMS_)	8.5 (10–4.7k)	5.23 (0.5–7k)	1.13 (0.1–500)	6.2/11	1
# of Rec. Channels	100	8	64	8	4
eural Stimulation					
Current Range (µA)	0–4.5 0–30 0–400	30	10–1350	1–127	250
Controllability	In-pixel 10 bit + 18 bits for chip range control In-pixel RAM for 30 independent current stimulus Global stimulation frequency control	N.A.	8 bit current DAC	7 bit current DAC	8 bit current DAC
# of Stim. Channels	100	1	64	2	4
